# The FRESHAIR4Life study: Global implementation research on non-communicable disease prevention targeting adolescents’ exposure to tobacco and air pollution in disadvantaged populations

**DOI:** 10.1038/s41533-024-00367-w

**Published:** 2024-06-04

**Authors:** Charlotte M. Hoffman, Anke Versluis, Sergiu Chirila, Bruce J. Kirenga, Amina Khan, Saima Saeed, Talant Sooronbaev, Ioanna Tsiligianni, D. K. Arvind, Linda C. Bauld, Floor A. van den Brand, Niels H. Chavannes, Hilary Pinnock, Pippa D. Powell, Jurjen van der Schans, Kamran Siddiqi, Siân Williams, M. J. J. Rianne van der Kleij

**Affiliations:** 1 Department of Public Health and Primary Care, Leiden University Medical Center, Leiden, The Netherlands; 2 Faculty of Medicine, Ovidius University of Constanta, Constanta, Romania; 3 Lung Institute & Department of Medicine, Makerere University, Kampala, Uganda; 4 The Initiative, Islamabad, Pakistan; 5 Indus Hospital and Health Network, Karachi, Pakistan; 6 National Center of Cardiology and Internal Medicine named after academician Mirrakhimov, Bishkek, Kyrgyzstan; 7 Department of Social Medicine, University of Crete, Rethymno, Greece; 8 School of Informatics, University of Edinburgh, Edinburgh, Scotland UK; 9 Usher Institute and SPECTRUM Consortium, University of Edinburgh, Edinburgh, UK; 10 Department of Family Medicine, Faculty of Health, Medicine and Life Sciences, Maastricht University, Maastricht, The Netherlands; 11 Allergy and Respiratory Research Group, Usher Institute, The University of Edinburgh, Edinburgh, UK; 12 European Lung Foundation, Sheffield, UK; 13 Unit of Global Health, Department of Health Sciences, University Medical Center Groningen, University of Groningen, Groningen, The Netherlands; 14 Department of Health Sciences, University of York, York, UK; 15 International Primary Care Respiratory Group, London, UK

**Keywords:** Disease prevention, Public health, Respiratory tract diseases, Health services

## Abstract

The FRESHAIR4Life study aims to reduce the non-communicable disease (NCD) burden by implementing preventive interventions targeting adolescents’ exposure to tobacco use and air pollution (AP) worldwide. This paper presents the FRESHAIR4Life methodology and initial rapid review results. The rapid review, using various databases and PubMed, aimed to guide decision-making on risk factor focus, target areas, and populations. It showed variable NCD mortality rates related to tobacco use and AP across the participating countries, with tobacco as the main risk factor in the Kyrgyz Republic, Greece, and Romania, and AP prevailing in Pakistan and Uganda. Adolescent exposure levels, sources, and correlates varied. The study will continue with an in-depth situational analysis to guide the selection, adaptation, and integration of evidence-based interventions into the FRESHAIR4Life prevention package. This package will be implemented, evaluated, assessed for cost-effectiveness, and iteratively refined. The research places a strong emphasis on co-creation, capacity building, and comprehensive communication and dissemination.

## Introduction

Non-communicable diseases (NCDs) are often preventable, yet they are the leading cause of death and disease burden worldwide^1^. Low- and middle-income countries (LMICs) and vulnerable populations in high-income countries^2,3^, further referred to as disadvantaged populations in this paper, are disproportionally affected^4^. To alleviate the increasing burden of NCDs and the mounting pressure they impose on healthcare systems, prevention of the most common NCD risk factors is urgently needed. As NCDs often develop as a result of prolonged and cumulative risk factor exposure, prevention of these risk factors should start early in life^5^.

Tobacco use and air pollution (AP) exposure are two of the most deadly NCD risk factors. In synergy, they account for approximately one-third of the NCD burden^6^. Evidently, the tobacco epidemic persists unabated. In 2020, 22.3% of the global population still used some form of tobacco, with populations in LMICs representing the vast majority (80%)^7^. The growing number of tobacco users, especially in LMICs, is fuelled by an ongoing intensive tobacco industry lobby targeting mainly young people and females^8^. After tobacco use, AP exposure is the second-leading cause of NCDs, causing 6.7 million premature deaths per year globally^6^. Nearly 90% of deaths attributed to ambient (outdoor) air pollution (AAP) occur in LMICs, where rapid industrialisation and urbanisation lead to significant emissions^9^. Moreover, one-third of the global population, mainly concentrated in LMICs, continues to rely on solid fuels such as wood, coal, and dung for cooking and heating^10^. This results in (additional) exposure to household air pollution (HAP).

Multi-level interventions are of vital importance for preventing NCD risk factors. Besides national-level policy statements and regulations, the World Health Organization (WHO) recognises that interventions on community and individual levels are inevitable for reducing risk factor exposure^11^. The WHO developed an implementation roadmap within its Global Action Plan on NCDs that calls for prioritising and upscaling ‘best buy’ interventions for NCD prevention^12^. Despite ample evidence of the effectiveness of these interventions, they are rarely successfully translated into practice^13^. Moreover, only a minority of community-based health intervention programs are implemented in LMICs, with even fewer specifically targeting younger populations like adolescents^14,15^.

Adolescence, defined as the phase of life from ages 10 to 21^16^, is a crucial developmental stage in which behaviours are formed, and health is increasingly understood and valued by the adolescents^17^. Moreover, adolescents’ growing interest in social- and societal topics empowers them to act as catalysts for promoting healthier lifestyles and driving positive transformations within their communities^18^. Therefore, starting with the implementation of preventive interventions targeting tobacco (use) and AP exposure during adolescence presents an ideal window of opportunity and could yield a higher return on investment^19,20^. However, as stated above, adolescents are often overlooked in NCD prevention efforts^21^.

The translational gap between NCD prevention and practice can be addressed through implementation science, which provides a structural approach to understanding the complexities of intervention implementation^22^. It pursues to determine why, how, for whom, and in which contexts interventions work, ultimately aiming to increase the effectiveness of interventions^23^. Particularly in LMICs, implementation science can help to prioritise cost-effective interventions and optimise the allocation of resources^24^.

In the FRESHAIR4Life (FA4Life) study, we aim to reduce the burden of NCDs due to tobacco (use) and AP exposure among adolescents in five countries – Greece, the Kyrgyz Republic, Pakistan, Romania, and Uganda – by optimising the implementation of tailored NCD preventive packages containing evidence-based interventions.

This 4-year Horizon Europe study, funded under the Global Alliance for Chronic Diseases (GACD) Life Course Research Programme, builds on the previously successful FRESH AIR projects^25^. While these projects focused mainly on diagnosing and treating chronic lung diseases, FRESHAIR4Life expands its scope to address NCDs more broadly, targeting its major risk factors for prevention.

The objectives of this paper are to:

1. Present the aim, objectives, participating countries and populations, and study phases of the FA4Life study;
2. Provide initial results of the first research phase, encompassing the existing knowledge on the burden of tobacco (use) and AP exposure among adolescents in the five countries, obtained through a rapid review;
3. Discuss the FA4Life study and highlight its potential future implications.

## Methodology: The FRESHAIR4Life study

### Aim & objectives

The FA4Life study aims to reduce the burden of NCDs by gaining knowledge, resources, and capacity to optimise the implementation of tailored preventive intervention packages, targeting adolescents’ exposure to tobacco (use) and AP in disadvantaged populations worldwide.

The six objectives in each of the participating countries are to:

1. Gain context-specific knowledge crucial for the selection, adaptation, and implementation of tobacco and/or AP-related preventive interventions;
2. Build and sustain dual capacity for NCD prevention through empowering youth advocates, fostering educational and healthcare providers’ leadership, and engaging family members^26^;
3. Co-create FA4Life prevention packages consisting of evidence-based interventions and develop tailored implementation strategies;
4. Iteratively implement, evaluate, and refine the FA4Life prevention packages;
5. Gather and disseminate evidence, resources, and tools for achieving sustainable and equitable implementation;
6. Inform on the effective scaling of the FA4Life packages and methods, including affordability.

### The FA4Life countries & populations

The study deliberately takes place in five diverse countries – Greece, the Kyrgyz Republic, Pakistan, Romania, and Uganda – and is carried out by a consortium of twelve organizations ranging from academic institutions to non-governmental organizations (Supplementary Table 1). All countries face a high burden of tobacco (use) and AP exposure, but their contexts differ substantially (Table 1)^27,28^. Despite being high-income countries, vulnerable populations exist in Greece and Romania. In both nations, a substantial risk of poverty disproportionately affects young people^29^. Greek youth have been severely affected by economic crises^3^, while Romania remains one of the poorest countries within the European Union^2^. Target groups that are involved include mid-to-late adolescents (14 to 21 years old), family members, providers (educators and healthcare professionals), and other stakeholders (e.g., public health managers, policymakers, and local authorities).

**Table 1:** Information on countries participating in FRESHAIR4Life, retrieved from The World Bank database^27^.

	Greece	Kyrgyz Republic	Pakistan	Romania	Uganda
Continent	Europe	Central Asia	South Asia	Europe	Africa
Surface area (square km x 1000)	132	200	796	238	242
Population size (millions)	10.6	6.80	236	19.0	47.2
Population age (median years)^28^	44.7	23.7	20.2	41.9	15.9
Income classification as assigned by the World Bank Group	High	Lower-middle	Lower-middle	High	Low
Life expectancy (years)	80.2	71.9	66.1	73.0	62.7
People living in urban areas (%)	80.0	37.5	37.7	54.5	26.2
Access to electricity (%)	100	99.7	94.9	100	45.2
Individuals using the internet (%)	78.5	77.9	21.0	83.6	10.3
Mobile cellular subscriptions (per 100 people)	110	130	81.6	119	65.7
Youth literacy (15–24 years old) (%)	99.2	99.8	72.0	99.4	89.4

### The phases of FA4Life

**Fig. 1:**
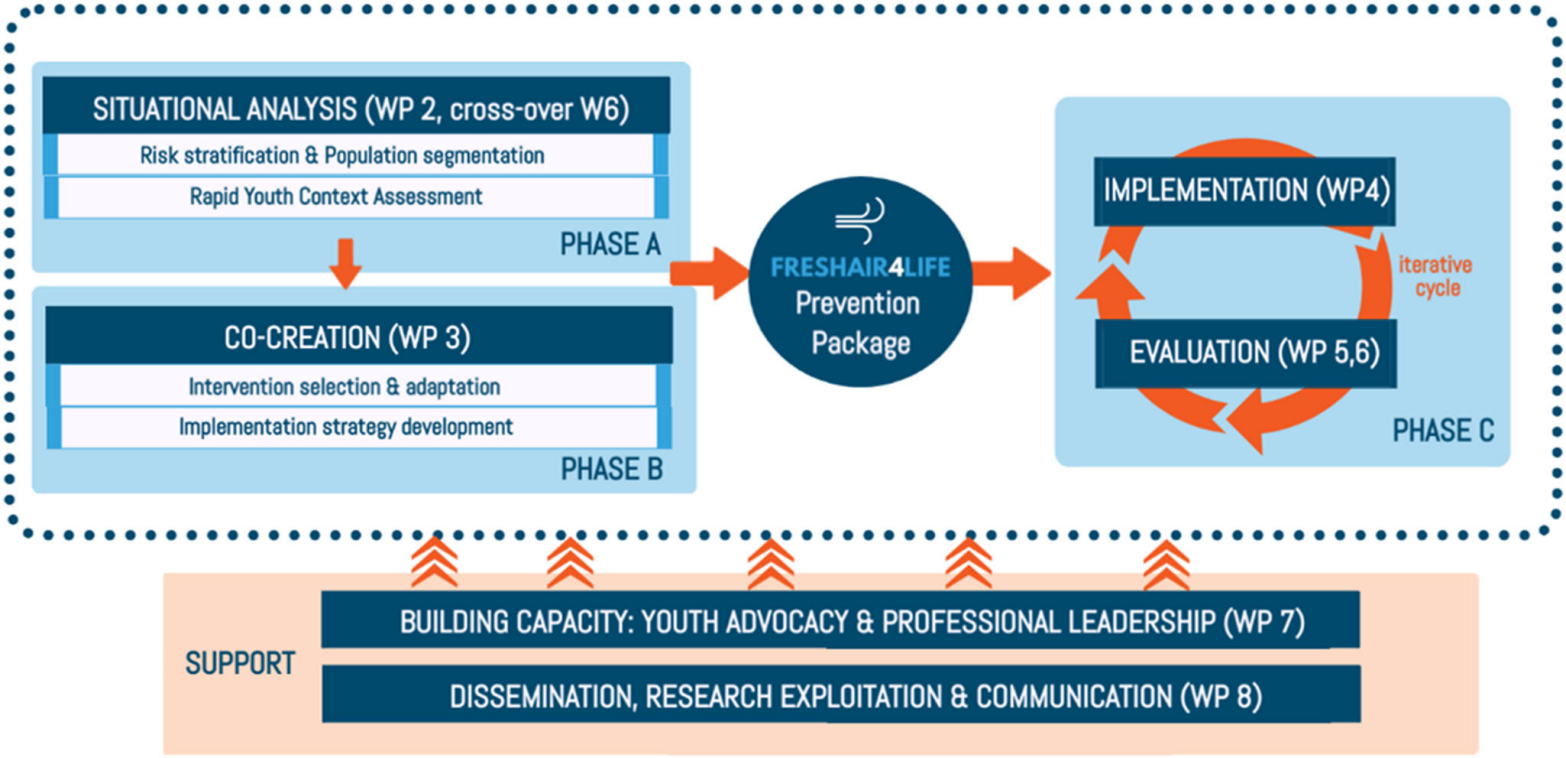
Study phases and work packages of FRESHAIR4Life.

FA4Life is a four-year implementation study comprising three phases and divided into eight work packages (WPs) (Fig. 1). A detailed overview of all WPs with their corresponding objectives and deliverables can be found in Supplementary Table 2.

The first phase includes a situational analysis, which guides the formation of preventive FA4Life packages and the choice of implementation strategies that are part of the second phase. In the third phase, these packages will be implemented and evaluated in iterative cycles. The Standards for Reporting Implementation studies of complex interventions (StaRI) guidelines will guide the research process^30^. In the following section, the different phases of the study are described in more detail.

#### Phase A: Situational analysis

*WP1* consists of coordinating tasks for the entire study’s duration. *WP2* involves a two-step situational analysis. The first step, a rapid review of adolescents’ risk factor prevalence in each participating country, has already been completed. Its results are presented in the *Results* section of this paper. The outcomes of the rapid review guided final decisions on the study site (within the country) and study population. The second step of the situational analysis will provide in-depth knowledge on the following context-specific topics: (1) perceived burden, (2) contextual drivers, (3) determinants of behaviour, and (4) (power) dynamics between stakeholders (Supplementary Figure 1). This knowledge will be crucial for selecting, adapting and implementing the interventions. Data on these determinants will be retrieved using a Rapid Youth Context Assessment (RYCA) based on the core principles of a rapid assessment process: (1) a community perspective, (2) rapid, in-depth iterative data collection and analysis, and (3) triangulation of data^31^. The RYCA has a mixed-methods design that includes qualitative interviews, focus group discussions, creative participatory research methods like *Photovoice*^32^, document analysis, and observation notes. Moreover, adolescents and providers will be invited to complete a quantitative questionnaire to triangulate qualitative results. Furthermore, personal exposure to airborne particulate pollutants will be measured in adolescents using the wearable Airspeck monitors^33^. A theoretical framework has been composed by combining three different frameworks (Supplementary Figure 2). This framework guided the design of data collection instruments and will facilitate structured data analyses.

#### Phase B: Intervention selection

**Fig. 2:**
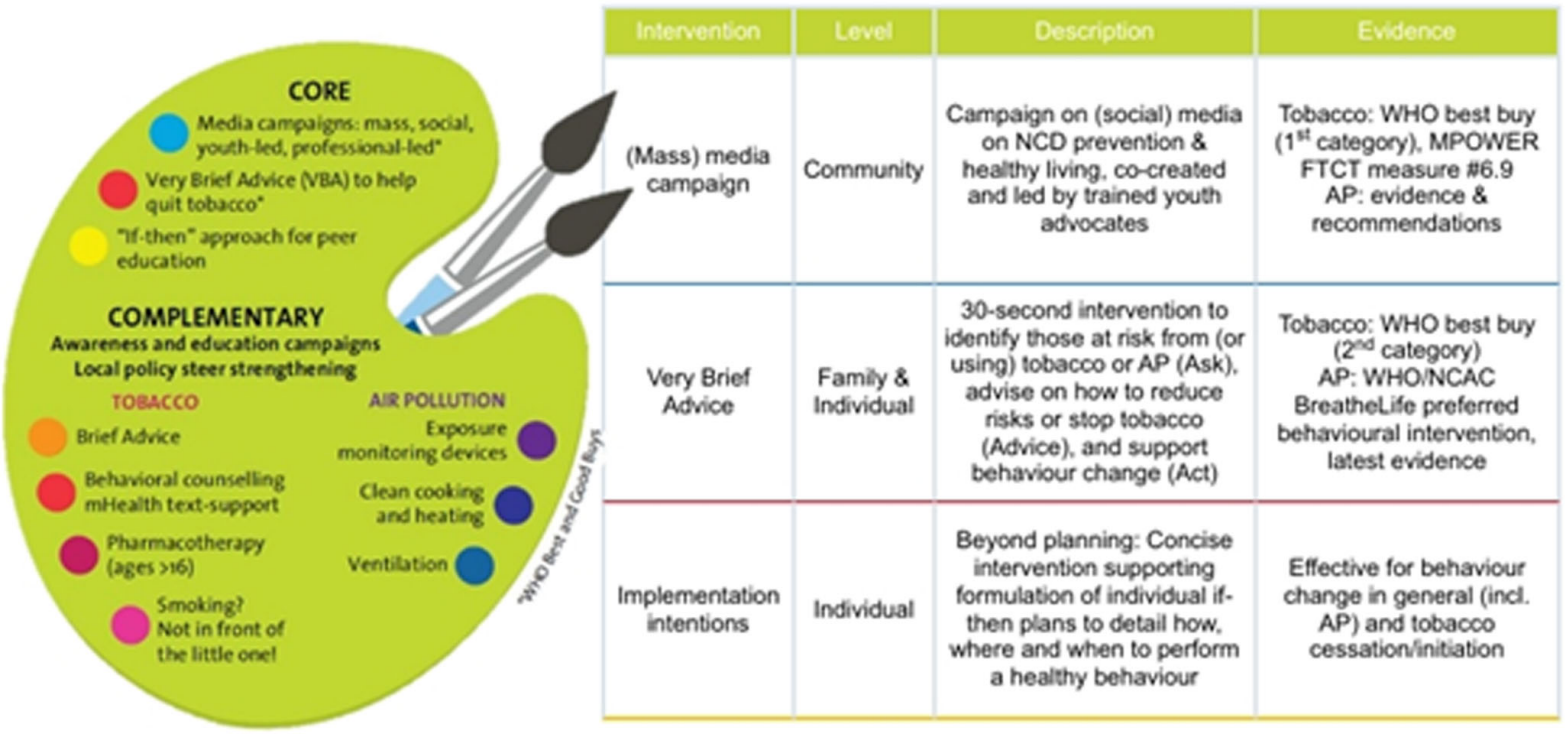
FA4Life Prevention palette and a short description of the FA4Life Core interventions.

The novel prevention palette method will be used to select interventions as part of *WP3* (Fig. 2). The palette lists tobacco and AP exposure prevention strategies proven to be (cost)effective, based on the WHO ‘best buys’^34^, FCTC MPOWER measures^35^, WHO global action plan for prevention and control of NCDs^12^ and relevant literature^20,36,37^. Each country must implement at least one of three core interventions (Fig. 2) complemented by additional interventions based on local needs. A detailed description of these interventions can be found in Supplementary Table 3. The chosen interventions will form the FA4Life prevention package and will be adapted in close collaboration with the local FA4Life team. Context-specific implementation strategies will be developed, drawing on the Expert Recommendations for Implementing Change (ERIC) project^38^.

#### Phase C: Intervention implementation, evaluation, and adaptation

**Fig. 3:**
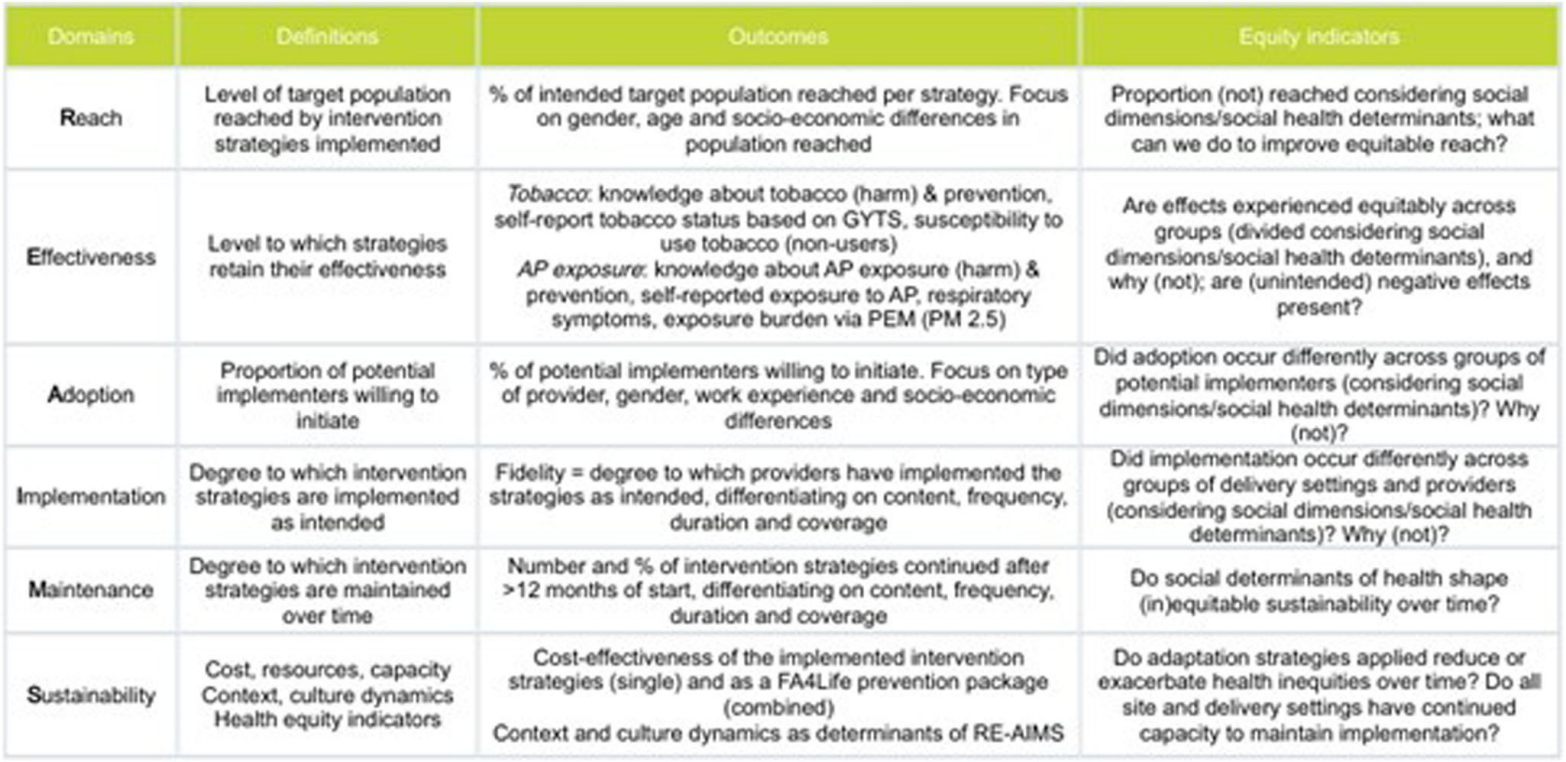
FRESHAIR4Life outcome dimensions across RE-AIMS domains.

The implementation and evaluation of the prevention packages will be done as part of *WP4*. The evidence-based tool of Proctor et al. (2013) will be used to prepare and plan for implementation^39^. Outcomes of implementation success will be evaluated and categorised into the RE-AIMS framework domains in *WP5*^40^. *Reach* and *Effectiveness* will be measured at the level of adolescents; *Adoption, Implementation*, and *Maintenance/Sustainability* on the provider level. Local teams will choose which dimensions to evaluate, including at least reach, implementation (fidelity), adoption, and one effectiveness parameter (Fig. 3). Health equity indicators will be considered during the evaluation process by performing a specific subgroup analysis for vulnerable groups. Finally, *WP6* investigates both the cost-effectiveness and the affordability of the FA4Life prevention packages using the widely applied socio-technical allocation of resources (STAR-)approach^41^.

#### Support throughout each phase

We aim to make a sustainable impact by emphasising co-creation, youth advocacy, and (research) capacity building throughout the study, as addressed in *WP7* and *WP8*. Co-creation will be ensured by uniting stakeholders in FA4Life teams at each site and engaging them in the design, execution, and evaluation processes.

##### Youth advocacy

Youth advocates will be trained, empowered, and supported through the FA4Life Digital Advocacy Champions initiative. Several adolescents from each country will enrol in a youth advocacy course and learn about NCDs, tobacco, and/or AP prevention and advocacy. After a group consultation to understand their needs and support efforts as peer supporters and community leaders, the adolescents will prepare, launch, and promote their own social media campaign.

##### Teach-the-teacher

Providers (healthcare workers and educators) will be supported in delivering interventions through a teach-the-teacher approach. After establishing an international master teaching faculty, a cohort of local master teachers will be recruited and trained on tobacco, and/or AP exposure prevention intervention strategies in adolescents and how to teach this curriculum to other providers.

##### Communication & dissemination

Risk factor-specific awareness will be raised by communicating tailored messages to target audiences on appropriate platforms (e.g., social media) and disseminating research findings to stakeholders and the general public. Eventually, all evidence, tools, and recommendations on implementing NCD prevention packages will be provided through an online, open-access FA4Life implementation toolbox on existing platforms.

## Results: Rapid review of tobacco (use) & AP exposure in the five FA4Life countries

This section provides the results of a rapid review of the burden of tobacco (use) and AP exposure among adolescents in the five FA4Life countries. This rapid review was conducted as the first step of the situational analysis belonging to *WP2*. A brief methods section, including aims, objectives, and search strategy for the rapid review has been provided in Box 1^42,43^.

Table 2 provides data retrieved from similar sources (e.g., from databases and outcomes of international surveys^44–47^), enabling cross-country comparisons. To contextualise the presented data, we provide an ancillary narrative summary based on identified common or notable factors (e.g., social, contextual) extracted from the literature.

**Table 2:** Overview of tobacco (use) and AP exposure among male and female adolescents in the five FA4Life countries.

	Greece		Kyrgyz Republic		Pakistan		Romania		Uganda	
Tobacco use/exposure										
**NCD death rate per 100.000 (age-standardised)**^44^	**107**		**133**		**147**		**114**		**41.4**	
(Male | female)	169	55.3	255	48.3	231	60.7	192	52.8	70.4	20.6
**Tobacco use prevalence (current), adolescents 13–15 years (%)**^45^	**14.1**		**6.0**		**10.7**		**14.6**		**10.5**	
(Male | female)	15.7	12.5	9.5	2.4	13.3	6.6	16.4	12.5	11.7	9.4
Cigarettes	**10.1**		**2.4**		**3.3**		**8.6**		**3.5**	
	10.3	9.9	4.2	0.6	4.8	0.9	9.8	7.3	4.7	2.4
Smokeless tobacco	**1.5**		**2.4**		**5.3**		**–**		**6.5**	
	1.9	1.1	4.3	0.6	6.4	3.7			7.1	6.0
E-cigarettes	**2.8**		**2.8**		**–**		**8.2**		**–**	
	3.9	1.7	3.9	1.7			10.1	5.9		
**Tobacco smoking prevalence adolescents 15–24 years, 2019 (%)**^46^	**30.4**		**13.8**		**7.01**		**28.4**		**5.55**	
(male | female)	36.4	30.4	23.4	3.98	11.9	1.83	34.3	22.3	7.80	3.36
**Trends in smoking prevalence adolescents 15–24 years, 1990–2019 (%)**^46^	**−25**		**−16**		−**40**		−**14**		**−4.6**	
(male | female)	−16	−36	−23	+71	−40	−17	−27	+24	−20	+65
**Mean age of initiation of tobacco use in the adult population (years)**^45^	**18.4**		**18.3**		**20.3**		**18.7**		**20.7**	
(male | female)	17.7	19.4	18.1	19.3	20.0	22.1	18.0	19.7	20.4	22.0
**SHS exposure (%)**^45^										
At home	57		17		21		36		19	
Public places (indoor)	67		14		38		34		30	

Air pollution exposure										
**NCD death rate per 100.000 (age-standardised)**^44^	**21.6**		**97.4**		**164**		**42.9**		**98.6**	
(male | female)	28.3	15.6	124	77.7	191	135	55.3	32.7	126	79.1
Ambient air pollution	**20.0**		**60.1**		**80.1**		**36.5**		**17.3**	
	26.3	14.4	81.5	44.5	101	58.9	48.2	27.0	25.7	11.4
Household air pollution	**0.14**		**33.4**		**76.4**		**6.05**		**80.5**	
	0.15	0.13	36.7	30.4	80.5	72.1	6.48	5.51	98.6	67.3
**PM2.5 annual mean concentrations (μg/m**^**3**^**)**^47^										
National	14.6		37.6		50.1		13.3		31.3	
Urban	15.6		39.7		51.6		14.1		32.3	
Cities	16.2		38.8		53.2		14.6		39.0	
Rural	12.7		36.2		48.5		12.2		29.5	
**Access to clean cooking alternatives (%)**^47^	100		76.6		49.3		87.7		0.50	
**Proportion of population with primary reliance on polluting fuels for cooking (%)**^47^	0		21.8		49.3		0		99.3	
Urban	0		5.0		12.8		0		98.5	
Rural	0		31.9		71.7		0		99.7	

*NCD* non-communicable diseases, *SHS* second-hand smoke.

Box 1 Rapid review: Aim, objectives & search strategy A rapid review was conducted to inform timely decisions regarding the choice of risk factors, target populations, and implementation sites for the FA4Life study. This type of review is defined as “a form of knowledge synthesis that accelerates the process of conducting a traditional systematic review through streamlining or omitting specific methods to produce evidence for stakeholders in a resource-efficient manner.”^42^ We have followed the interim guidance provided by the Rapid Reviews Methods Group for reporting our methodology and data^43^.

**Aim**

To synthesize evidence on tobacco (use) and AP exposure among adolescents in the five FA4Life countries, aiming to establish a strong foundation for informed decision-making concerning risk factors, target populations, and implementation sites.

**Objectives**

- Compare the burden of tobacco (use) and AP exposure within and between the participating countries;
- Identify at-risk adolescents in terms of age, gender, socioeconomic status, and other related correlates;
- Determine regions (i.e., urban or rural) within countries with significantly high burdens of tobacco (use) and AP exposure;
- Provide insights and recommendations for decision-making regarding risk factors (i.e., tobacco use and/or AP exposure), target populations, and implementation sites.

**Search strategy**

We identified and assessed various large (routine) databases that consist of tobacco and/or AP data (e.g., Global Health Observatory, Global Burden of Disease repository, UNICEF MICS) and most recent outcomes of (inter)national surveys, e.g., Global Adult Tobacco Survey (GATS), Global Youth Tobacco Survey (GYTS), Demographic and Health surveys (DHS), WHO STEPS Noncommunicable Disease Risk Factor Surveillance and Eurobarometer. An overview of the assessed data sources, with indications of their respective years of the most recent version per country, is provided in Supplementary Table 4.

Additionally, in March 2023, a literature search was performed in PubMed after consultation with a certified librarian for articles published between 2000 and the present, using MeSH terms, free search terms, and synonyms for “Adolescents” AND (“Tobacco use” OR “Air pollution”) AND the name of the country. A full-text filter was applied. This search yielded 441 (Greece), 24 (Kyrgyz Republic), 447 (Pakistan), 158 (Romania), and 113 (Uganda) articles. Articles were considered relevant if they met the following inclusion criteria:

1. Data on adolescents or specific risk groups; *and*
  - Reported prevalence rates or related indicators of risk factor burden, *or*
  - Addressed potential correlates (e.g., gender, socioeconomic status, educational level) of the risk factors; *and*
2. Presented country-specific data, i.e., of Greece, the Kyrgyz Republic, Pakistan, Romania, and/or Uganda.

Title and abstract screening, full-text assessment, and data extraction were performed separately by two researchers. A total of 30 (Greece), 7 (Kyrgyz Republic), 33 (Pakistan), 20 (Romania), and 24 (Uganda) articles were used for this review. Due to time constraints, dual screening, quality verification, or a systematic risk-of-bias assessment of publications were not undertaken.

### Comparison of Global Burden of Disease Study results

**Fig. 4:**
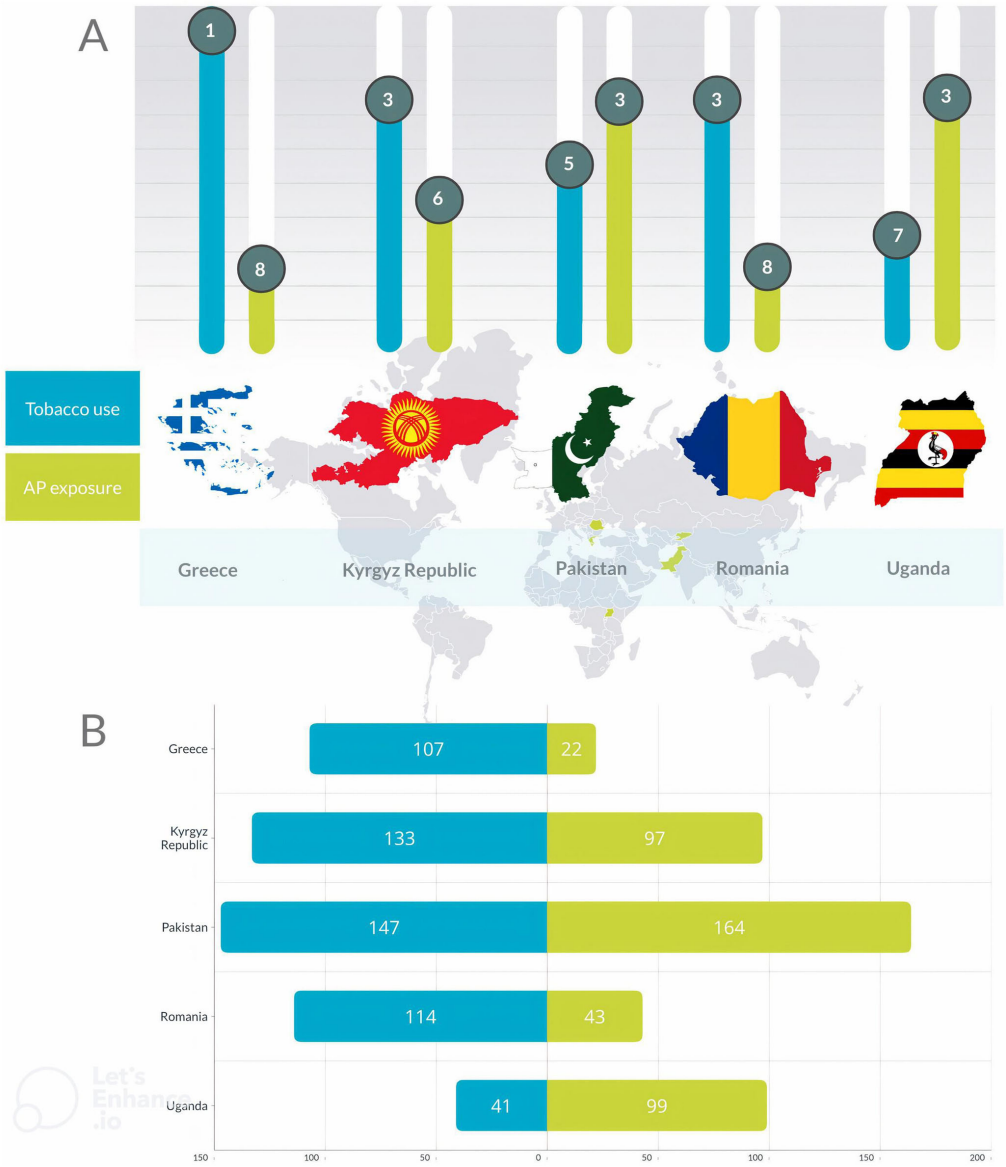
Impact of tobacco use and air pollution on non-communicable disease death rates in the five FA4Life countries. **A** Place of tobacco use and air pollution exposure in the top 10 ranking of risk factors contributing to the non-communicable death rate per country; **B** Non-communicable death rate per 100.000 (age-standardised) as a result of tobacco use and air pollution exposure per country^44^. NCD non-communicable diseases.

Tobacco use and AP exposure are among the ten key risk factors for death caused by NCDs in all five countries (Fig. 4)^44^. Tobacco use is the most important cause of NCD deaths in Greece, Romania, and the Kyrgyz Republic, whereas AP exposure contributes mostly to NCD deaths in Pakistan and Uganda (Table 2 and Fig. 4). The highest death rate for both tobacco use and AP is seen in Pakistan. Rates are generally higher for males than females. Among females in the Kyrgyz Republic, Pakistan, and Uganda, AP contributes to a higher NCD death rate than tobacco, with ambient AP being the predominant form in the Kyrgyz Republic and household AP in the latter two countries^44^.

### Tobacco use and exposure

#### Tobacco use rates & trends

The highest prevalence rates of tobacco use are seen in adolescents from Greece and Romania (Table 2). Tobacco smoking prevalence among adolescents aged 15–24 years between 1990-2019 has decreased in males for all countries but increased relatively and in absolute numbers, though not significantly, for females in the Kyrgyz Republic and Uganda^46^. In contrast to smoking tobacco, the prevalence rates of smokeless tobacco (SLT) have only shown a marginal reduction in Pakistan (i.e. a reduction of 0.15% in females and 0.07% in males) since 1990 over the course of 30 years^48^. This suggests that control efforts are primarily focused on and/or impact smoking tobacco rather than all forms of tobacco.

#### Gender & age

In all countries, tobacco use rates are higher for males than for females. These differences are less explicit in Greece^49–52^ and Romania^53,54^, a pattern commonly observed in high-income countries^55^. Moreover, some studies indicate even higher tobacco use rates for young females in Greece^52,56^. The low tobacco use rates among females in the Kyrgyz Republic and Pakistan may be attributable to cultural norms, i.e., the social disapproval of females who smoke^57,58^. The age of initiation ranges between 18 and 21 years old in all countries^46^. Remarkably, despite this age in Pakistan being almost the highest among the five countries, the age of initiation among adolescents is considerably low (40% initiate tobacco use <10 years of age)^45^.

#### Socioeconomic status (SES) & Education

Tobacco use is common among lower socioeconomic groups in Pakistan^59^ (DHS 2018, UNICEF MICS 2019-2020) and Uganda^60–62^ (DHS 2016), less pronounced in Greece^63,64^ and Romania^65,66^. The Kyrgyz Republic forms an exception, where higher wealth and education correlate with increased cigarette use; however, SLT is more common among the less educated (DHS 2012). A lack of knowledge on the adverse health effects of tobacco^67–72^, low health literacy^73,74^, and illiteracy in general^75–77^, play an important role in tobacco use. The latest GYTS results show that the percentage of adolescents that are educated about the dangers of tobacco at school ranges from merely 50% in Pakistan to 77% in Uganda^45^.

#### Contributing factors

Frequently associated correlates of tobacco use that are applicable in all countries include parents, siblings, and/or friends who use tobacco^50,53,63,75,78–91^, a low educational level of the parents^83,92^, risky concomitant behaviours such as alcohol^56,60,68,93–97^ and drug use^98^ and the existence of negative emotions and psychological symptoms^68,99–102^. Obesity or being overweight is frequently associated with tobacco use in Romania and Uganda^103–105^ and is inversely correlated in Greece, especially in females^106,107^. Interestingly, females in Greece^108^, Romania^109^, and Pakistan^70^ believe that tobacco use contributes to weight control.

#### Geography

Pakistan (GATS 2014, DHS 2018) and Uganda (GATS 2013, DHS 2016) have higher rates of tobacco use in rural areas. However, males in these countries tend to live in urban areas^59,61^. No significant differences are known in Romania^110^. In Greece, students in (semi-)urban areas smoke more often than students in rural areas^56,63^. The largest proportion of tobacco users in the Kyrgyz Republic live in urban areas (i.e., Bishkek and Osh) and the Chûy region in particular (DHS 2012).

#### Forms of tobacco products

In Greece and Romania, conventional cigarettes are the predominant form of tobacco. Among adolescents in Pakistan and Uganda, SLT is the most popular, with similar prevalence rates observed in the Kyrgyz Republic (Table 2). Risk factors for SLT are similar to other forms of tobacco use^111,112^. In Pakistan, especially females use SLT^113–115^; in adolescents, up to 54% of tobacco users consume SLT^114^. Heat-not-burn products (HTPs) and Electronic Nicotine Delivery Systems (ENDS) like e-cigarettes and vapes are emerging as smoking alternatives. Studies in Greece^79,116,117^, Romania^89,118–122^ and Pakistan^123–125^ indicate a greater susceptibility among younger individuals, a strong association with conventional tobacco use, and the belief that these alternatives are less harmful and assist in smoking cessation. Waterpipe, also known as shisha or hookah, is mainly popular among youth in Pakistan^126–129^, the Kyrgyz Republic^130^ and Uganda^131^, and is often used for the same reasons as HTPs/ENDS.

#### Second-hand smoke (SHS) exposure

Greek adolescents exhibit the highest exposure to SHS (Table 2) and the lowest support (56%) for prohibiting smoking in outdoor public places, despite the highest knowledge on SHS health risks of all five countries (77%)^45^. A study in Uganda shows high support for prohibiting smoking in outdoor public places (up to 99%), associated with higher education, awareness of SHS harm, a smoke-free home, and exposure to antismoking media^132^. Knowledge of SHS health risks among adolescents is lowest in Romania and Uganda (55–57%)^45^. SHS exposure was more common in rural households compared to urban in Pakistan^133,134^ and Uganda^135,136^.

#### Smoking cessation

Approximately 60% of adolescents between 13-15 years old tried to quit smoking in the past 12 months, with Uganda as an exception with a rate of 93%^45^. A notable difference is seen in the proportion of adolescents receiving help for smoking cessation in Greece and Romania (12–14%) compared to the other countries (30%)^45^.

### Air pollution exposure

All countries exhibited significantly high concentrations of fine particulate matter (PM2.5), a standard proxy indicator for AP, above the WHO air quality guideline level of 5 μg/m^3^ (Table 2). This is especially the case in the Kyrgyz Republic, Pakistan, and Uganda. AAP is the predominant risk factor in Pakistan, HAP in Uganda, and both AAP and HAP in the Kyrgyz Republic^44^.

#### Ambient AP

According to IQ Air, Pakistan was the third most polluted country in the world between 2018-2022^137^, with all of its inhabitants living in areas where the exposure levels exceed the WHO guideline^138^. Here, AP exposure is the leading risk factor for all-cause, all-age mortality among both sexes^139^. In the Kyrgyz Republic, the average annual PM pollution has tremendously increased^140^. Most AAP originates from vehicular emissions and domestic heating, the latter contributing to deteriorating exposure levels in winter due to increased biomass burning. A study involving 4100 students in Pakistan demonstrated considerable adverse effects on both physical and psychological health due to AP^141^. Additionally, AP was identified as a significant predictor of hospital visits for respiratory health symptoms in Pakistan^142,143^, Romania^144,145^, and Greece^146–148^. In Greece, adverse (health) consequences of desert dust storms are recently receiving increased academic attention^146,149^. A limited number of Ugandan studies considered AAP. However, a recent study conducted in Kampala revealed increased AP exposure levels, which exhibited an independent association with atherosclerotic disease in HIV-positive adolescents^150^.

#### Household AP

Merely 0.5% of the Ugandan population has access to cleaner cooking alternatives. Solid fuel use is also still common in Pakistan and the Kyrgyz Republic (Table 2), mainly in rural areas, and is often associated with lower SES and low education (UNICEF MICS 2018, 2019-2020). Previous FRESH AIR studies have shown significant exposure to HAP and high prevalence rates of COPD in rural communities in the Kyrgyz Republic and Uganda^151,152^. Additionally, in Uganda, HAP was independently associated with asthma and persistent (childhood) cough^153–155^. Females and children are mostly exposed to HAP^156,157^. Notably, a retrospective study in Ugandan lung cancer patients shows that patients are mainly female never-smokers^158^. Solid fuels for heating are still used in Greece and Romania despite available alternatives. In Romania, approximately a quarter of the children have an iron stove in their bedroom, and the same proportion of homes have fireplaces^159^. Results from FRESH AIR in Greece showed that 61% of rural households used solid fuel for heating, resulting in PM2.5 levels exceeding WHO guidelines^57^. Half of the households owned clean fuel devices, but their usage depended on financial reasons and was influenced by Greece’s austerity measures^160^.

### Decisions on risk factor focus, adolescent populations, and implementation sites

In conclusion, this rapid review established a strong foundation for decision-making regarding risk factor focus, at-risk adolescents, and implementation sites in each participating country. It shows that significant disparities exist in the burden of tobacco use and AP across and within countries. As expected, high-income countries primarily face behavioural risk factors, like tobacco use, while LMICs increasingly face AP as an environmental issue. Both risk factors rank among the leading causes of NCD mortality in all five countries. Tobacco use is a prevalent concern in scientific literature, particularly in high-prevalence countries, whereas studies on AP are still limited despite its severity, as indicated by recent air quality measurements. Amid the context of climate change, there is a growing interest in AP and its impact on human health (planetary health). This growing interest has increased academic output on this topic in Pakistan but remains scarce in the Kyrgyz Republic.

Risk stratification to identify adolescents at risk is challenging due to a lack of comparability between local studies regarding populations, regions, and associated contributing factors. Our literature search often reported inconsistent findings of at-risk adolescents, burdened areas, and other potential correlates. Moreover, the scarce available evidence for specific factors contributing to an increased burden of tobacco use and AP in certain countries (e.g., the influence of SES on tobacco use in the Kyrgyz Republic), and relatively overrepresented study areas (e.g., Lahore and Karachi in Pakistan), make it difficult to draw conclusions. Additionally, while the burden on females appears less than that for males, it is essential to recognise the narrowing gap in tobacco use prevalence between genders and the disproportionate impact of HAP on females’ health. While evolving trends may not be fully evident yet, it is imperative to proactively implement preventive interventions to avert potential escalation in future numbers for both genders equally. What stands out, however, is the double burden of tobacco (use) and AP exposure faced by low SES populations.

Considering all these factors, we have adopted a broad scope, including diverse adolescents (e.g., in terms of gender, age, and SES) and regions (Supplementary Table 5). Both tobacco (use) and AP exposure are prevalent and pose challenging problems across all five countries. Therefore, we will include both risk factors for the in-depth situational analysis (step 2 of *WP2*) in all study sites. Only in Uganda, where AP causes a disproportionate burden compared to tobacco use and the cultural unacceptability of tobacco complicates thorough research, will the primary focus be on investigating AP. To ensure representativeness, the research will encompass both urban and semi-urban or rural areas, as risk factors are often unevenly distributed between these regions. Urban areas have been selected as starting points due to feasibility, safety, familiarity, existing connections in the population, and available monetary and human resources. Leveraging existing networks makes this approach the most favourable option for the study.

## Discussion: FRESHAIR4Life’s next steps and implications

Building upon the knowledge, experiences, and capacity acquired from prior FRESH AIR projects, FRESHAIR4Life continues its efforts to combat NCDs in disadvantaged populations globally. Previous FRESH AIR research contributed significantly to increased awareness, improved diagnosis, and recognition of chronic (lung) diseases in low-resource settings while pioneering the implementation scope^25^. The high burden of NCDs, with tobacco use and AP exposure as major contributors, underscores the urgent need for prevention in disadvantaged populations. This need formed the foundational rationale of FRESHAIR4Life. Initiating preventive interventions during adolescence was considered an optimal window of opportunity.

This research will place a strong emphasis on the implementation of evidence-based interventions on a small scale in a variety of contexts. In harmony with the principles of implementation science, each WP is designed on widely accepted frameworks and theories. Crucial in implementation science is an understanding of the context. To that end, the rapid review has provided a strong foundation for decision-making on implementation sites and populations as an important first step. In these implementation sites, an in-depth situational analysis will be conducted together with local teams and the involvement of stakeholders. The selected interventions will then be implemented through existing local networks, as we recognize that effective and sustainable implementation can only be achieved with collaborative, multi-level, and transdisciplinary efforts. Importantly, we will adapt existing, evidence-based interventions as recommended by the WHO instead of developing new interventions from scratch.

FRESH AIR’s strength, and therefore pre-eminence in this new study lie in its co-creation and capacity building with a strong bottom-up approach. On that account, community engagement and soliciting stakeholder support are considered vital^161^. Participatory research, especially in adolescents, offers an excellent opportunity to increase this engagement^162^. Hence, we pursue adapting to adolescents’ engagement with digital tools and social media by incorporating *Photovoice* as a method in our situational analysis and using (social) media platforms in our communication and dissemination strategies. To ensure capacity building on the provider and stakeholder side, the teach-the-teacher approach has proven successful in various studies, including previous FRESH AIR projects^25^. Finally, a multifaceted communication and dissemination strategy will promote upscaling, reaching broader audiences, and maximising impact.

Our study’s structured project approach applies to all five countries, ensuring comparability while allowing content-level adjustments to suit specific contexts. Within the diverse range of populations across countries, each with eminent cultures, beliefs, and practices, our objective is to identify similarities and overarching strategies. By doing so, we strive to create solutions that can be extrapolated and applied effectively in various other settings worldwide.

Ultimately, we expect that the outcomes of FA4Life will lead to:

- Improved knowledge, awareness, and healthy behaviours of adolescents and families relevant to NCD prevention;
- Establishment of (improved) local health policies on NCD (risk) prevention, specifically on tobacco and AP exposure, in adolescents;
- Improved capacity of providers, managers, and policymakers to promote, implement, and lead NCD prevention;
- Integration of FA4Life results and tools in local guidelines and quality frameworks;
- Implementation knowledge, resources, and tools to support researchers, providers, managers, and other relevant (community) stakeholders to develop, implement, and sustain NCD prevention programs;
- Reduced risk factor prevalence, leading to a future reduction in premature NCD mortality.

### Supplementary information

FRESHAIR4Life Study - Supplementary File
