# Supplementary material for: The FRESHAIR4Life study: Global implementation research on non-communicable disease prevention targeting adolescents’ exposure to tobacco and air pollution in disadvantaged populations
Source: NPJ Prim Care Respir Med. 2024 Jun 4;34:14. doi: 10.1038/s41533-024-00367-w (PMC11150571; doi:10.1038/s41533-024-00367-w)
Supplement: Supplementary file 1 — FRESHAIR4Life Study - Supplementary File [file 41533_2024_367_MOESM1_ESM.pdf]

Supplementary file

Belonging to **The FRESHAIR4Life study: Global implementation research on non-communicable disease prevention targeting adolescents' exposure to tobacco and air pollution in disadvantaged populations**

Contents

Supplementary Table 1. Consortium members of FRESHAIR4Life 2

Supplementary Table 2. Description of Work Packages, Objectives & Deliverables for FRESHAIR4Life 3

Supplementary Figure 1. Determinants of Work package 2: Situational analysis 5

Supplementary Figure 2. Theoretical framework developed for FRESHAIR4Life WP2: Situational analysis 6

Supplementary Table 3. Description of the FA4Life core and complementary interventions 8

Supplementary Table 4. Assessed resources per country for the rapid review 10

Supplementary Table 5. Decisions on risk factors and target areas per country 11

Supplementary Table 1. Consortium members of FRESHAIR4Life

Institution	Type of organisation	Country	Lead
**Leiden University Medical Center** *(coordinator)*	Academia/medical center	The Netherlands	Dr. Rianne van der Kleij
**International Primary Care Respiratory Group**	Clinically led charitable organisation	United Kingdom	Ms. Sian Williams
**Makerere University**	Academia/medical center	Uganda	Dr. Bruce Kirenga
**Ministry of Health Kyrgyz Republic**	Ministry of health	Kyrgyz Republic	Prof. Talant Sooronbaev
**University of Crete**	Academia/medical center	Greece	Dr. Ioanna Tsiligianni
**National Centre for studies in Family Medicine**	Foundation	Romania	Dr. Cristina Isar
**European Lung Foundation**	Patient-led organisation	Belgium	Dr. Pippa Powell
**University of Edinburgh**	Academia/medical center	United Kingdom	Prof. Hilary Pinnock
**University of Maastricht**	Academia/medical center	The Netherlands	Prof. Onno van Schayk
**University of York**	Academia/medical center	United Kingdom	Prof. Kamran Siddiqi
**University Medical Center Groningen**	Academia/medical center	The Netherlands	Prof. Maarten Postma
**The Initiative**	NGO	Pakistan	Dr. Amina Khan

Supplementary Table 2. Description of Work Packages, Objectives & Deliverables for FRESHAIR4Life

WP	Title	Objectives & tasks	Deliverables (month due)
1	Project management	• To ensure the FA4LIFE project is managed both efficiently and effectively, and that all project objectives, milestones and deliverables are achieved.	D1.1 Project management plan delivered (M4)<br>D1.2 Data Management Plan delivered / updated (M4, updated at M12 & 24)<br>D1.3 Progress report delivered (M18, 36)
2	Situational analysis	• Identify those adolescents most in need or at risk through population segmentation & risk stratification, mindful of context, location, gender, age, and equity indicators;<br>• Gain insight into the needs and perceptions towards NCD prevention in the adolescent population identified;<br>• Deliver recommendations for intervention selection and implementation strategy development.	D2.1 Advisory report on which region, topic, population, and site to target (M8)<br>D2.2 Scientific publication on results of situational analysis across and within countries (M28)<br>D2.3 Report with recommendations for adaptation- and implementation strategies (M14)
3	Co-creation of FA4Life prevention packages	• Select and adapt intervention strategies following situational analysis results;<br>• Develop at least 3 tailored implementation strategies per prevention package;<br>• Secure resources necessary to implement the FA4LIFE prevention packages in each site.	D3.1 Scientific publication on the use of the palette prevention method to select and adapt interventions (M30)<br>D3.2 Report on the FA4LIFE prevention packages developed in each site (M20)
4	Implementation of FA4Life prevention packages	• Deliver implementation protocols for the FA4LIFE prevention packages;<br>• Implement the FA4LIFE prevention packages using tailored implementation strategies developed;<br>• Establish an implementation toolbox comprising knowledge, tools and resources gained.	D4.1 Protocols for the implementation of the prevention package in each site (M23)<br>D4.2 Scientific publication comparing co-creation and implementation across sites (M42)<br>D4.3 FA4LIFE Implementation toolbox online available (M36, update M48)
5	Evaluation of FA4Life prevention packages	• Select which RE-AIMS dimension(s) to evaluate and what outcomes to measure;<br>• Develop evaluation methods and materials and plan data collection at each demonstration site;<br>• Assess Reach, Effectiveness, Adoption, Implementation, Maintenance and Sustainability;<br>• Identify implementation determinants relevant to Adoption, Implementation, and Maintenance.	D5.1 Local protocols for the evaluation of the FA4LIFE prevention package (M24, update M28)<br>D5.2 Scientific publication reporting on RE-AIMS outcomes for prevention packages across sites (M46)<br>D5.3 Scientific publication reporting on implementation determinants influencing RE-AIMS outcomes (M48)

**6**	Health economics & data-drive solutions	• Assist intervention selection by analysing affordability and 'value for money' using the STAR approach;<br>• Assess the health economic burden of tobacco and AP exposure prevention;<br>• Assess the cost-effectiveness and scale-up budgets of the prevention packages;<br>• Use sensor-based frameworks to assess AP exposure burden and intervention effectivity.	D6.1 Report on the health economic burden of tobacco and AP exposure in adolescents in each site (M32)<br>D6.2 Scientific publication on sensor-based framework to assess (M42)<br>D6.3 Budget impact report of scale-up of cost-effective interventions per country (M48)
**7**	Building dual capacity: youth advocacy & professional leadership	• Establish youth advocacy at each site through the FA4LIFE Digital Advocacy Champions Initiative;<br>• Support, teach and empower FA4LIFE teams at each site (cross-connection with task 2.2);<br>• Recruit providers and managers to NCD prevention leadership school and ongoing mentoring programme;<br>• Identify potential motivators and incentives for sustained preventative action.	D7.1 Curriculum and educational materials package in local languages and adapted to the local situation (M26)<br>D7.2 Report on Digital Advocacy Champions initiative to empower youth advocacy on NCD prevention (M40)<br>D7.3 Curriculum for leadership development and cohort of leaders established (M24, M36)<br>D7.4 Digital guide on how to implement three-tier teaching and learning programme (M40)
**8**	Dissemination, research exploitation & communication	• Disseminate our experiences, understanding of implementation research and tobacco and AP exposure prevention in adolescents in disadvantaged populations, frameworks, resources and tools;<br>• Develop effective public information and awareness-raising campaigns;<br>• Deliver the FA4LIFE case for action including necessary policy steps.	D8.1 Dissemination, exploitation and communication plan delivered (M6, 18, 36)<br>D8.2 Project identity package with website, social media platforms and infographics (M6,18, 36)<br>D8.3 FA4LIFE case for action delivered (M24, M36, M48)

Supplementary Figure 1. Determinants of Work package 2: Situational analysis

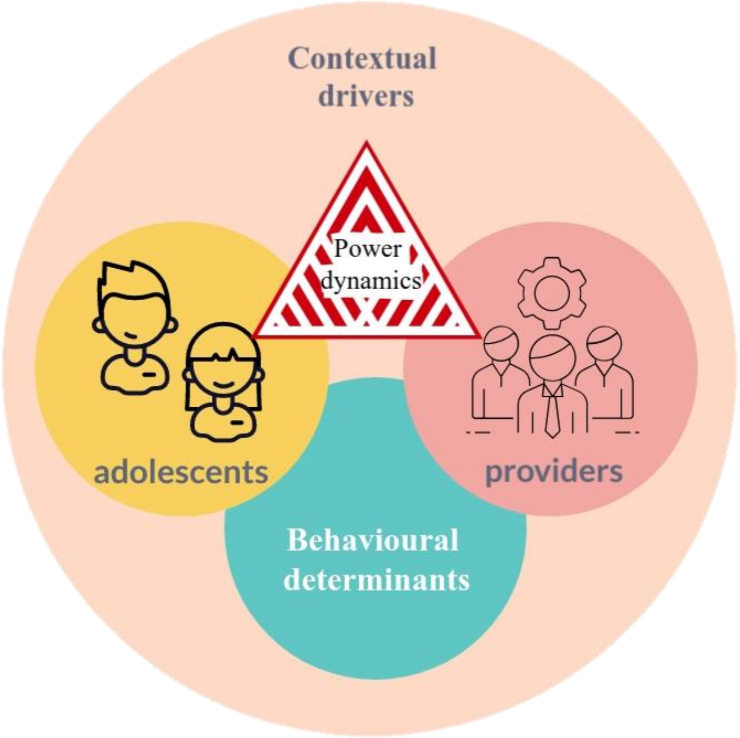

Burden: the combined impact of biological, psychological and social factors as result of exposure to AP and/or tobacco use

Contextual driver: factors that shape the context and impact implementation (e.g. socio-cultural, economic, political)

Behavioural determinant: antecedents to behaviour that precede and provide the rationale, motivation, or drive for behaviour (change) (e.g. knowledge, motivation, beliefs about consequences)

Power dynamics: culturally-specific interpersonal interactions between individuals or groups that involve the exercise of influence, control, and authority, shaping the distribution of power within a social system

Supplementary Figure 2. Theoretical framework developed for FRESHAIR4Life WP2: Situational analysis

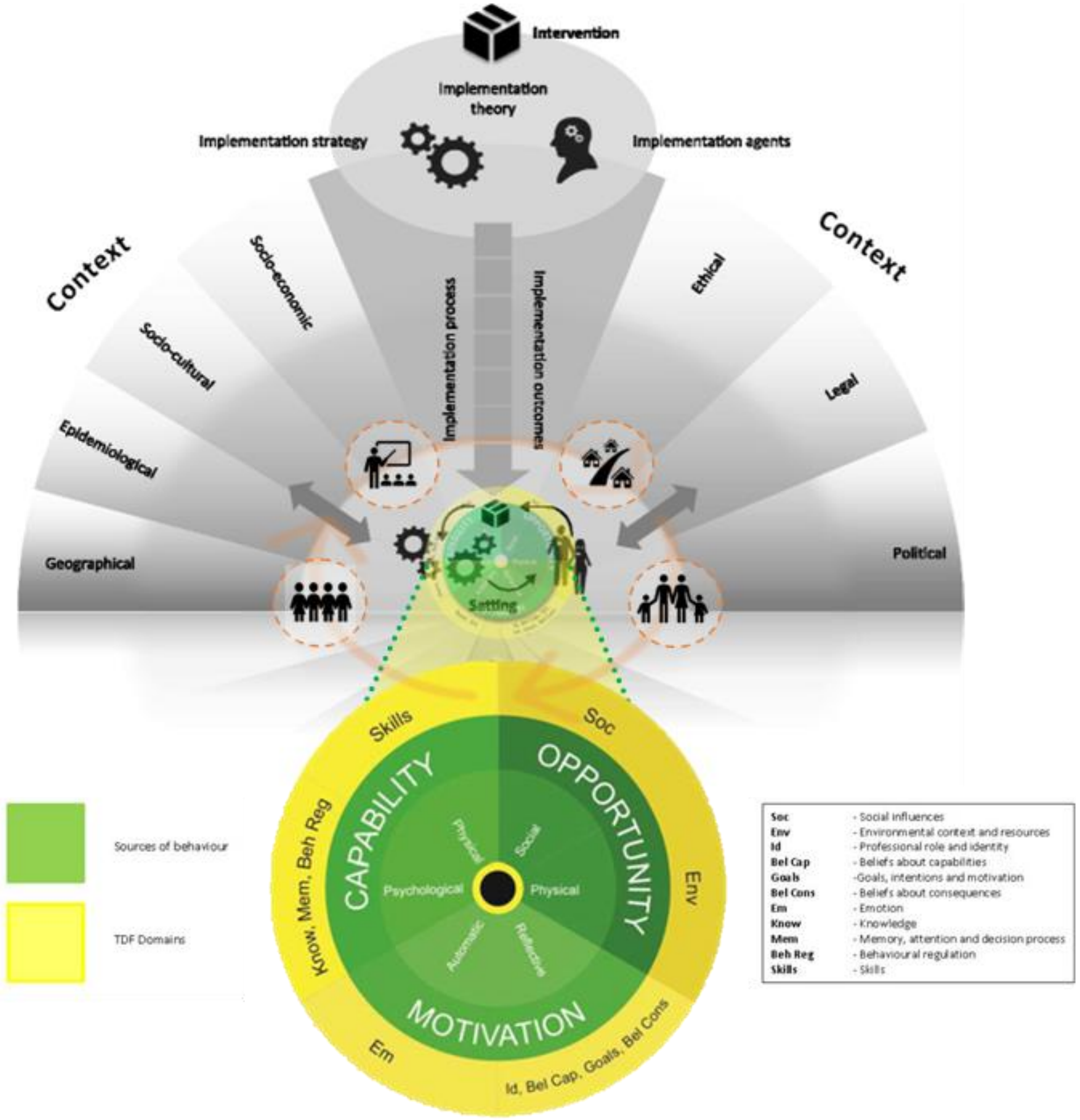

We use a theoretical framework as a structured approach to identify relevant context characteristics and for interpretation and contextualisation of the collected data. The framework helps furthermore to theoretically underpin material development. The theoretical framework used in this study is a combination of the Context, Input, Components, and Outcomes (CICI) framework[1] and the Theoretical Domains Framework (TDF),[2] with a special focus on the key elements of The Protection Motivation Theory (PMT).[3] To emphasize the importance of the adolescents' social environment for context-assessment and behaviour change – i.e. their family, peers, school and community – we display these roles prominently and distinctively within our combined theoretical framework.

CICI: to map and analyse contextual drivers relevant to implementation of tobacco and/or AP preventive interventions

The CICI framework is a comprehensive approach that aims to simplify and structure complexity of implementation contexts to advance the understanding of whether and how interventions work. It helps to map and analyse relevant contextual factors (e.g. geographical, socio-cultural, legal, political) on different levels (i.e. macro, meso and micro) that may influence the risk factor burden and behaviour, or facilitate/hamper the implementation of preventive interventions. This framework guided the development of data collection instruments (e.g. topics for interviews and FGD) to assess contextual drivers and helped to identify relevant stakeholders. After data collection, we will use the CICI framework to systematically analyse the results of the situational analysis and to subsequently inform intervention selection and implementation strategies.

TDF: to assess individual factors for facilitating/hampering behaviour change

The Theoretical Domains Framework (TDF) is a framework used to understand and address the barriers and facilitators to behaviour change. It aims to identify the key theoretical domains that influence behaviour and to map these onto behaviour change techniques that can be used to change behaviour. It provides a useful and indispensable addition to the CICI framework – that focuses on the broader context – by offering a deeper understanding of the adolescents' and providers' behaviour relating to risk factor exposure and implementation of preventive interventions, and identifying factors that can be barriers or facilitators for behaviour change within each theoretical domain. The data collection tools are developed to provide insight into the applicable domains (e.g. knowledge, beliefs about capabilities and consequences, social influences) and identify relevant determinants of behaviour change, which can ultimately help to guide intervention selection, adaptation and implementation.

PMT: to highlight important elements of risk assessment and preventive behaviour

Lastly, The Protection Motivation Theory (PMT) is a theoretical framework that explains how individuals make decisions about engaging in protective behaviour. It suggests that individuals are motivated to protect themselves from harm by weighing the perceived threat against their ability to cope with the threat. Since FA4Life focuses on the perception of risk factors and the adoption of preventive interventions, this framework will help in assessing adolescents' perceived threat of the risk factor exposure, as well as their perceived ability to cope with these threats. While TDF provides a broader insight into behaviour change, and has also incorporated elements of PMT, we use this specific framework to highlight auxiliary elements of the PMT within the TDF (e.g. perceived vulnerability, perceived severity, self-efficacy).

Supplementary Table 3. Description of the FA4Life core and complementary interventions

	Description	Evidence	Application
**Core interventions**			
**(Mass) media campaign** *featuring youth advocates*	Mass media, such as television, radio, printed materials and social media can reach a large audience and increase population-level awareness, attitude- and behaviour change at relatively low cost.	(Mass) media campaigns that educate the public about the harms of tobacco and second-hand smoke is an important WHO 'best buy.'[4] Additionally, (youth-led) campaigns have been shown effective at influencing a broad selection of health-related behaviours, including promoting modern cookstove use, limiting tobacco use in adolescents and air pollution exposure reduction.[5-9]	A (mass) media campaign will be built and launched in each site following the results from the FA4LIFE Digital Advocacy Champions initiative and stories of those that matter through Photovoice and digital stories
**Very brief advice (VBA)**	Provision of a 30-second intervention by practitioners to identify those at risk or in need (Ask), advise on how to reduce risks or stop unhealthy behaviours (Advice), and support corresponding behaviour change (Act).	Feasible and cost-effective to support change in a variety of health behaviours,[10] e.g. for preventing tobacco uptake and supporting cessation.[11,12] For tobacco use it is recognized as WHO 'best buy'.[4] For AP exposure, VBA has not yet been validated.	VBA will be taught in a cascading teach-the-teacher approach, teaching providers on how to deliver this intervention at family and individual level.
**IF-THEN implementation intention**	Concise intervention supporting formulation of individual if-then plans to detail how, where, and when to perform a healthy behaviour.	Meta-analyses indicate strong effects of implementation intentions on behaviour change, particularly for goals that individuals are motivated to pursue, and effectiveness in various adolescent risk behaviours including smoking initiation.[13,14]	This intervention will be implemented at schools and delivered by schoolteachers.
**Complementary interventions**			
**Personal exposure monitoring (PEM)**	Airspeck monitors measure personal exposure to air borne particulates (PM 10, PM 2.5 and PM 1), temperature and relative humidity. This monitor will generate real-time data that gives a deeper insight into the burden of AP exposure and tobacco use/exposure at	The device has been used successfully by a variety of subjects (including healthy subjects, asthmatics, COPD patients and COVID-19 recovered subjects) in both high and lower-middle income countries.[15]	Measurements will be performed to assess exposure burden in the first phase and intervention strategy effectiveness in phase 3. Data obtained will feed a mass-media campaign with youth advocates.

	individual level in different (micro) environments.		
**Clean cooking & heating**	Advancing cleaner cooking and heating methods is vital to reduce HAP. Involving adolescents as future cookstove stakeholders can be a launch pad for promoting adoption and sustained use of improved technologies.	In FRESH AIR, but also other projects performed by FA4LIFE consortium members, clean heating and cookstoves solutions were successfully implemented.[16]	Educate adolescents and families on traditional cooking's impacts, exposure risks, clean cooking solutions and maintenance. FA4Life teams will take the lead in cleaner cooking initiatives through partnering with local networks, utilising the Cleaner Cookstove Implementation Tool (CleanCIT) and Clean Fuel Implementation Tool (CleanFIT).[17]
**Behavioural support interventions**	Behavioural support, including (group) counselling sessions, that aim to assist individuals in modifying their behaviours, habits, and attitudes related to tobacco use, ultimately increasing their chances of successfully quitting.[18]	A meta-analysis of data from nine RCTs supports the effectiveness of behavioural interventions (including counselling, education, acupuncture, hypnosis, laser therapy) to support smoking cessation among school-aged children and adolescents.[19]	Suitable and effective interventions are context- and resource dependent. Training as well as willingness and time to deliver the intervention on an ongoing or repeated basis may be required. Decisions will be made in consultation with FA4Life teams.
**Pharmacotherapy**	Medications approved to help quit smoking by reducing cravings and withdrawal symptoms, such as nicotine replacement therapy (NRT), bupropion and varenicline. All of these three are on the WHO Model Lists of Essential Medicines	NRT is an effective intervention in LMIC settings.[20] Data for varenicline in adolescents is less robust.[21] Pharmacotherapy may be more effective with behavioural interventions, at least among adult smokers.[22]	This intervention is highly context-dependent as not all pharmacotherapies for the treatment of tobacco dependence are available in all countries.
**Smoking? Not in front of the little one!**	Education programme based on the health belief model and principles of motivational interviewing for training providers to communicate with parents - including adolescents, such as teen parents - about second-hand smoke exposure in children <5.	Proved highly effective: after implementation, the percentage of parents or caretakers that smoked in the presence of their children decreased from 41% (before implementation) to 18% (3 years post implementation).[23]	The choice for implementing this intervention is dependent on the adolescent population that will be chosen as intervention group.

Supplementary Table 4. Assessed resources per country for the rapid review

Source	Greece	Kyrgyzstan	Pakistan	Romania	Uganda
Both risk factors					
**Demographic and Health surveys**	n/a	2012	2018	n/a	2016
**Global Health Observatory**	2019	2019	2019	2019	2019
**World Bank Open Data**	2021	2021	2021	2021	2021
**Global Burden of Disease repository**	2019	2019	2019	2019	2019
**UNICEF MICS**	n/a	2018	2019-2020	n/a	n/a
Tobacco use					
**WHO Global Youth Tobacco Survey (GYTS)**	2013	2019	2013	2017	2018
**WHO Global Adult Tobacco Survey (GATS)**	2013	n/a	2014	2018	2013
**Health Behaviour in School-aged Children (HBSC)**	2017-2018	n/a	n/a	2017-2018	n/a
**Global school-based student health survey (GSHS)**	n/a	n/a	2009	n/a	2003
**WHO STEPS Noncommunicable Disease Risk Factor Surveillance**	n/a	2013	2014	n/a	2014
**Eurobarometer survey**	2020	n/a	n/a	2020	n/a
Air pollution					
**WHO Air Pollution Portal**	2019	2019	2019	2019	2019
**IQAir**	2021	2021	2021	2021	2021

n/a = not available

Supplementary Table 5. Decisions on risk factors and target areas per country

	Urban	Peri-urban / Rural
**Tobacco use**	Greece; Kyrgyz Republic; Pakistan; Romania	Greece; Kyrgyz Republic; Pakistan; Romania
**Ambient air pollution**	Greece; Kyrgyz Republic; Romania; Uganda	Kyrgyz Republic; Pakistan; Romania; Uganda
**Household air pollution**	Kyrgyz Republic; Uganda	Greece; Kyrgyz Republic; Pakistan; Uganda

References

1 Pfadenhauer, L. M. *et al.* Making sense of complexity in context and implementation: the Context and Implementation of Complex Interventions (CICI) framework. *Implementation Science* **12**, 21, doi:10.1186/s13012-017-0552-5 (2017).

2 Atkins, L. *et al.* A guide to using the Theoretical Domains Framework of behaviour change to investigate implementation problems. *Implement Sci* **12**, 77, doi:10.1186/s13012-017-0605-9 (2017).

3 Rogers, R. W. & Prentice-Dunn, S. in *Handbook of health behavior research 1: Personal and social determinants.* 113-132 (Plenum Press, 1997).

4 World Health, O. Tackling NCDs: 'best buys' and other recommended interventions for the prevention and control of noncommunicable diseases. (World Health Organization, Geneva, 2017).

5 Wakefield, M. A., Loken, B. & Hornik, R. C. Use of mass media campaigns to change health behaviour. *Lancet* **376**, 1261-1271, doi:10.1016/s0140-6736(10)60809-4 (2010).

6 Evans, W. D. *et al.* Evaluation of Behavior Change Communication Campaigns to Promote Modern Cookstove Purchase and Use in Lower Middle Income Countries. *Int J Environ Res Public Health* **15**, doi:10.3390/ijerph15010011 (2017).

7 Riley, R. *et al.* How do we effectively communicate air pollution to change public attitudes and behaviours? A review. *Sustainability Science* **16**, 2027-2047, doi:10.1007/s11625-021-01038-2 (2021).

8 Carson, K. V. *et al.* Mass media interventions for preventing smoking in young people. *Cochrane Database Syst Rev* **6**, Cd001006, doi:10.1002/14651858.CD001006.pub3 (2017).

9 Valdez, E. S. *et al.* Youth Participatory Action Research for Youth Substance Use Prevention: A Systematic Review. *Substance Use & Misuse* **55**, 314-328, doi:10.1080/10826084.2019.1668014 (2020).

10 Anokye, N. K., Lord, J. & Fox-Rushby, J. Is brief advice in primary care a cost-effective way to promote physical activity? *Br J Sports Med* **48**, 202-206, doi:10.1136/bjsports-2013-092897 (2014).

11 Hum, A. M., Robinson, L. A., Jackson, A. A. & Ali, K. S. Physician communication regarding smoking and adolescent tobacco use. *Pediatrics* **127**, e1368-1374, doi:10.1542/peds.2010-1195 (2011).

12 Hollis, J. F. *et al.* Teen reach: outcomes from a randomized, controlled trial of a tobacco reduction program for teens seen in primary medical care. *Pediatrics* **115**, 981-989, doi:10.1542/peds.2004-0981 (2005).

13 Gollwitzer, P. M. & Sheeran, P. in *Advances in Experimental Social Psychology* Vol. 38 69-119 (Academic Press, 2006).

14 Conner, M. *et al.* Effectiveness and cost-effectiveness of repeated implementation intention formation on adolescent smoking initiation: A cluster randomized controlled trial. *J Consult Clin Psychol* **87**, 422-432, doi:10.1037/ccp0000387 (2019).

15 Arvind, D. K., Maiya, S. & Sedeño, P. A. in *2021 IEEE 17th International Conference on Wearable and Implantable Body Sensor Networks (BSN).* 1-4.

16 van Gemert, F. *et al.* Effects and acceptability of implementing improved cookstoves and heaters to reduce household air pollution: a FRESH AIR study. *npj Primary Care Respiratory Medicine* **29**, 32, doi:10.1038/s41533-019-0144-8 (2019).

17 Boudewijns, E. A. *et al.* Facilitators and barriers to the implementation of improved solid fuel cookstoves and clean fuels in low-income and middle-income countries: an umbrella review. *The Lancet Planetary Health* **6**, e601-e612, doi:10.1016/S2542-5196(22)00094-8 (2022).

18 Hartmann-Boyce, J. *et al.* Behavioural interventions for smoking cessation: an overview and network meta-analysis. *Cochrane Database Syst Rev* **1**, Cd013229, doi:10.1002/14651858.CD013229.pub2 (2021).
19 Peirson, L., Ali, M. U., Kenny, M., Raina, P. & Sherifali, D. Interventions for prevention and treatment of tobacco smoking in school-aged children and adolescents: A systematic review and meta-analysis. *Prev Med* **85**, 20-31, doi:10.1016/j.ypmed.2015.12.004 (2016).
20 Akanbi, M. O. *et al.* The efficacy of smoking cessation interventions in low- and middle-income countries: a systematic review and meta-analysis. *Addiction* **114**, 620-635, doi:10.1111/add.14518 (2019).
21 Gray, K. M. *et al.* High-dose and low-dose varenicline for smoking cessation in adolescents: a randomised, placebo-controlled trial. *Lancet Child Adolesc Health* **4**, 837-845, doi:10.1016/s2352-4642(20)30243-1 (2020).
22 Stead, L. F., Koilpillai, P., Fanshawe, T. R. & Lancaster, T. Combined pharmacotherapy and behavioural interventions for smoking cessation. *Cochrane Database Syst Rev* **3**, Cd008286, doi:10.1002/14651858.CD008286.pub3 (2016).
23 Crone, M. R., Reijneveld, S. A., Willemsen, M. C. & Hira Sing, R. A. Parental education on passive smoking in infancy does work. *European Journal of Public Health* **13**, 269-274, doi:10.1093/eurpub/13.3.269 (2003).
